# Development and evaluation of a water‐profile‐based calibration method for 2D ionization chamber arrays in medical linear accelerator quality assurance

**DOI:** 10.1002/acm2.70830

**Published:** 2026-09-25

**Authors:** Kihong Pak, Sung‐woo Kim, Byungchul Cho, Jungwon Kwak, Seonghoon Jeong, Kyeongyun Park, Jisoo Kim, Jonghan Park, Si Yeol Song, Seonyeong Noh, Jongin Oh, Seokjin Kang, Chiyoung Jeong

**Affiliations:** ^1^ Department of Radiation Oncology Asan Medical Center Songpa‐gu Seoul Republic of Korea; ^2^ Department of Radiation Oncology, Asan Medical Center University of Ulsan College of Medicine Songpa‐gu Seoul Republic of Korea; ^3^ Graduate School of Convergence Science Korea University Seongbuk‐gu Seoul Republic of Korea

**Keywords:** 2D ionization chamber array, beam profile, IC profiler, quality assurance, water‐scanner profile based calibration

## Abstract

**Purpose:**

To develop and evaluate a water‐profile‐based (WP) calibration method for two‐dimensional (2D) ionization chamber arrays used in medical linear accelerator (linac) quality assurance (QA), and to compare its accuracy, efficiency, and cross‐energy applicability with the manufacturer‐provided wide‐field (WF) calibration method.

**Methods:**

Beam profiles were measured using an IC Profiler (ICP) and a 3D water scanner on three TrueBeam linacs (TB1–TB3) at five photon energies (4, 6, 10 MV flattened; 6, 10 MV flattening‐filter‐free [FFF]) with a 20 × 20 cm^2^ field size at a depth of 10 cm. A total of 240 profiles were acquired across eight independent measurement sessions. The WP method derived adjusted calibration factors (ACFs) by directly matching ICP detector responses to water‐scanner reference profiles, and was evaluated under three strategies: (i) machine‐specific (single linac reference), (ii) machine‐integrated (reference pooled across three linacs), and (iii) cross‐energy applicability across 20 inter‐energy combinations. Performance metrics included per‐detector relative differences (mean absolute error [MAE] and standard deviation [SD]) and beam profile characterization (symmetry and flatness). Paired *t*‐tests (*n* = 30) compared the machine‐integrated WP method with the WF method.

**Results:**

The WF method yielded a per‐detector MAE of 0.31%, with symmetry and flatness MAEs of 0.49% and 0.44%, respectively. The WP method significantly reduced these uncertainties to 0.15–0.16% (per‐detector), 0.21–0.27% (symmetry), and 0.27–0.28% (flatness), with medium‐to‐large effect sizes (Cohen's *d* = 0.55–0.99; all *p* < 0.01). Cross‐energy application of WP‐derived ACFs yielded a per‐detector MAE of 0.25%, higher than that of energy‐specific WP calibration but lower than that of the WF method in this dataset. Total calibration time decreased from approximately 75 min (WF) to 10–15 min (WP).

**Conclusion:**

For the tested 20 × 20 cm^2^ field on TrueBeam linacs with a CC13/Blue Phantom2 water‐scanner reference, the proposed WP calibration method provided closer agreement with the water‐scanner reference and a substantially shorter calibration time than the manufacturerprovided WF method. Machine‐integrated calibration is recommended for routine multi‐linac implementation, while energy‐specific ACFs should be used whenever feasible to maximize accuracy.

## 1 INTRODUCTION

One of the primary responsibilities of a qualified medical physicist is ensuring the dosimetric accuracy of medical linear accelerators (linacs) through rigorous quality assurance (QA). Conventionally, beam profiles and beam quality are characterized using three‐dimensional (3D) water scanning systems during beam commissioning and periodic (monthly or annual) QA. While 3D water scanners serve as the conventional reference instrument for beam‐profile characterization, their setup is labor‐intensive and time‐consuming. In recent years, two‐dimensional (2D) ionization chamber arrays, such as the IC Profiler (ICP; Sun Nuclear Corporation, Melbourne, FL, USA, software version 3.5), have been widely adopted for routine QA, beam steering, and even commissioning.^1^, ^2^, ^3^, ^4^, ^5^ These arrays consist of ionization chambers arranged in horizontal, vertical, and diagonal axes, allowing for the instantaneous acquisition of beam profiles with a single irradiation. Compared to 3D water scanners, 2D arrays offer significant advantages in terms of setup efficiency due to their compact size, lighter weight, and ease of alignment with the linac's crosshairs and treatment room lasers.

However, a critical prerequisite for using 2D ion chamber arrays is the accurate calibration of individual detector sensitivities. Because the inherent sensitivity of each chamber varies, they must be cross‐calibrated to ensure a uniform response across the array. Manufacturers typically provide standardized calibration protocols; for instance, the ICP utilizes the Wide Field (WF) method, which involves multiple irradiations with various rotations and shifts to determine the Adjusted Calibration Factors (ACFs). Despite its utility, the WF method may not fully account for the machine‐specific beam characteristics of a particular linac, as it relies on a generalized geometry‐based algorithm. Furthermore, the sensitivity of detectors can change over time, necessitating a more robust and clinically relevant calibration approach. To address these limitations, a calibration strategy that directly maps the ICP detector responses to the beam profiles measured by a 3D water scanner—hereafter referred to as the Water‐Profile (WP) method—has been proposed.

A similar concept was previously introduced by Shen et al.^6^ as the Central Field (CF) method for the ICP‐MR in an MR‐linac environment. The CF method derives detector‐specific correction factors by directly comparing array measurements to water‐scanner reference profiles. In the present work, we adopt the term WP method to more explicitly reflect the use of water‐scanner profiles as the calibration reference. Their study, however, was conducted on a single MR‐linac system with a single beam energy, leaving unexplored the effects of reference data grouping strategy (e.g., machine‐specific vs. machine‐integrated calibration) and cross‐energy applicability of the calibration factors—factors that are particularly relevant for conventional linac environments where multiple machines and energies must be managed. In this study, we provide a comprehensive evaluation of the WP method, with a particular focus on the impact of different reference data grouping strategies and its cross‐energy applicability. By comparing the WP method with the manufacturer‐provided WF method, we aim to demonstrate that this approach provides closer agreement with the water‐scanner reference profiles acquired for WP calibration, together with a substantially shorter calibration time.

## 2 MATERIALS AND METHODS

### 2.1 Beam profile measurement

#### 2.1.1 Reference data acquisition using a 3D water scanner

Reference beam profiles were obtained from three TrueBeam (TB) linear accelerators (Varian Medical Systems, a Siemens Healthineers Company, Palo Alto, CA, USA). These linacs provide nominal photon energies of 4, 6, and 10 MV for flattened beams, and 6 and 10 MV for flattening‐filter‐free (FFF) beams, with a maximum field size of 40 × 40 cm^2^ at a source‐to‐surface distance (SSD) of 100 cm.

The water‐scanner reference profiles were acquired specifically for WP calibration in this study, using a 3D water scanning system (Blue Phantom2) equipped with CC13 ionization chambers (sensitive volume 0.13 cc; inner radius 3.0 mm, active length 5.8 mm) and myQA Accept software version 8.3 (all from IBA Dosimetry GmbH, Schwarzenbruck, Germany). The beam profiles were acquired in both inline and crossline directions at a depth of 10 cm. Although annual QA at our institution typically evaluates a 30 × 30 cm^2^ field, a 20 × 20 cm^2^ field size was selected for this study to ensure compatibility with the active area of the ICP (32 × 32 cm^2^) and the dimensions of the buildup slab phantoms (30 × 30 cm^2^). The slab phantoms were composed of RW3 water‐equivalent material (PTW, Freiburg, Germany; physical density 1.045 g/cm^3^). Accordingly, the validity of the WP‐derived ACFs demonstrated in this study is limited to the 20 × 20 cm^2^ calibration field; application to other field sizes, including the 30 × 30 cm^2^ field used for annual QA, requires separate validation.

A total of eight independent measurement sets were performed across the three linacs (TB1–TB3), with each set comprising five beam energies (4, 6, 10 MV flattened and 6, 10 MV FFF) measured in both inline and crossline directions, yielding 240 individual beam profiles in total. Three measurement sets were dedicated to generating the calibration files, while the remaining five were used for evaluation. To incorporate potential setup uncertainties into the analysis, the 3D water scanner was completely independently set up and re‐aligned for each measurement session. Prior to each measurement session, central‐axis (CAX) alignment of the 3D water scanner with respect to the linac beam was corrected using the CAX correction feature of myQA Accept: inline and crossline profiles acquired at depths of 5 and 25 cm were used to derive the lateral shift and angular tilt of the scanner, which were then applied to all subsequent profile acquisitions within the session. For analysis, all profiles—from both the water scanner and the ICP—were normalized to the CAX dose value. The CAX position of each water‐scanner profile was determined using the scanning software's built‐in center‐finding algorithm (midpoint of the 50% dose level on each side of the profile), while for the ICP the CAX was defined as the response of the central detector (detector index 0).

To ensure independence between calibration and evaluation, the eight measurement sets were partitioned as follows: the first measurement set from each linac (TB1–TB3) was used exclusively to determine the ACFs (either machine‐specific or machine‐integrated), while the remaining five sets (three sets from TB1, and one set each from TB2 and TB3) were reserved for performance evaluation. No measurement session was used for both calibration and evaluation, ensuring that the reported metrics reflect the method's generalization performance rather than its fitting accuracy on the training data.

#### 2.1.2 Beam profile measurement using an ICP

**FIGURE 1:**
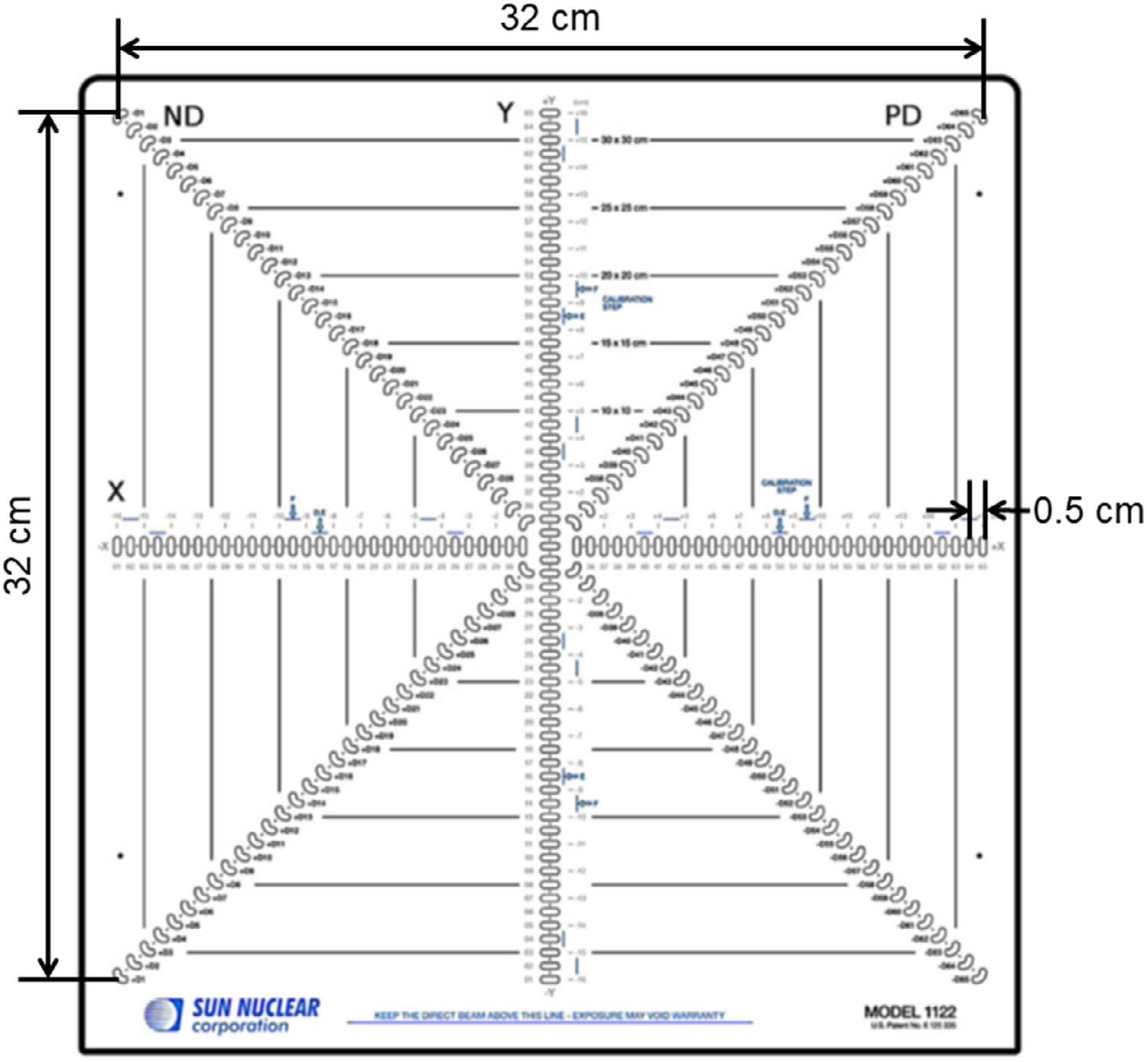
The beam's eye view of ICP device. The shapes and locations of ion chambers are described on the panel with labels for each detector and axis. The blue arrows indicate the alignment for the manufacturer's calibration procedures.

The ICP was employed as the 2D detector array. The ICP features 251 ionization chambers (individual volume of 0.05 cc) arranged along the X, Y, and diagonal axes. Specifically, 65 chambers are spaced at 0.5 cm intervals along the Y axis, and 63 chambers along the X axis (note: two chambers in X axis adjacent to the central axis are omitted). The total active area of the array is 32 × 32 cm^2^.^7^ Figure 1 shows the beam's eye view image of the ICP. To ensure that the beam characteristics remained constant during comparison, ICP measurements were performed immediately following each water‐scanner measurement. RW3 slabs with a physical thickness of 9.1 cm were placed on the ICP surface; together with the inherent 0.9 cm buildup of the ICP, this constituted the nominal 10‐cm measurement depth. The field size was set to 20 × 20 cm^2^ at an SSD of 100 cm, with the SSD set at the upper surface of the RW3 slab stack placed on the ICP using the optical distance indicator (ODI). This setup corresponded to the water‐phantom measurement geometry of SSD 100 cm at a depth of 10 cm. At each measurement session, a 2D MV image of the ICP was acquired, and the translational and rotational alignment of the array was matched to the orthogonal crosshairs passing through the image center; residual translational and rotational differences were < 0.5 mm and < 0.1°, respectively. To minimize mechanical variations such as couch sag, the longitudinal and lateral couch coordinates were fixed at 140 and 0 cm, respectively, throughout the study, and the vertical position was set using the ODI. The raw signal distributions along the X and Y axes were exported for analysis, while diagonal data were excluded from this study.

#### 2.1.3 Beam profile characterization metrics: symmetry and flatness

For all subsequent analyses, the in‐field region was defined as ± 80% of the half field width, determined at the 50% dose level of the measured profile; a tolerance of ≥40% of the CAX dose was applied at the field edge to accommodate the discrete 5 mm detector spacing of the ICP. The in‐field region is indicated on the representative profile in Figure 3. This definition is applied consistently to the symmetry, flatness, and per‐detector relative‐difference analyses described below. The beam symmetry and flatness, used as the primary beam profile characterization metrics throughout this study, are defined as follows. The symmetry is defined as the maximum point‐difference symmetry within this in‐field region, computed over the detector pairs symmetric about the central axis:

$$\mathrm{Symmetry}=\frac{D_{j}-D_{sym,j}}{D_{CAX}}\times 100\left(\%\right)$$

where $D_{j}$ is the dose at detector j on the positive half‐axis (right for the crossline profile, superior for the inline profile), $D_{sym,j}$ is the dose at the geometrically symmetric detector about the central axis, and $D_{CAX}$ is the dose at the central axis. For each profile, j corresponds to the detector at which the absolute deviation $|D_{j}-D_{sym,j}|$ is maximal within the in‐field region; the signed value of this maximum‐deviation pair is reported as the symmetry. By this convention, a negative symmetry value indicates that the negative half‐axis side of the profile (left for crossline, inferior for inline) exhibits the higher dose, while a positive value indicates the opposite. The flatness is defined over the same in‐field region as:

$$\mathrm{Flatness}=\frac{D_{\max}-D_{\min}}{D_{\max}+D_{\min}}\times 100\left(\%\right)$$

where $D_{max}$ and $D_{min}$ denote the maximum and minimum doses.

### 2.2 Calibration methods

This section describes the calibration methods evaluated in this study. Table 1 summarizes the four approaches—the manufacturer's wide‐field (WF) method and the three variants of the proposed water‐profile (WP) method—together with their corresponding evaluation strategies. Detailed procedures are presented in the following sub‐sections.

**TABLE 1:** Overview of calibration methods and corresponding evaluation strategies.

Category	Method	Machine	Calibration Strategy	Evaluation Process	Reference dataset
Manufacturer Method	Wide Field	TB2	Manufacturer's six‐measurement sequence (rotation and shifts)	Validation using independent measurement data from TB1–TB3	TB2 manufacturer six‐measurement data
Proposed Method	Machine‐Specific	TB1	Matching of reference ICP data to the corresponding water‐scanner profile	Comparison of subsequent TB1 ICP measurements against water‐scanner profiles	Single‐linac TB1 WP reference
	Machine‐Integrated	TB1‐3	Matching averaged ICP data to averaged water‐scanner profiles	Performance test using ICP measurements from all three linacs	Pooled TB1–TB3 WP references
	Cross‐energy applicability	TB1	Calibration derived from a single beam energy on TB1 (same approach as Machine‐Specific)	Assessment of ACF robustness by applying calibration factors derived from a different beam energy	Single‐energy TB1 WP reference (applied across energies)

#### 2.2.1 Manufacturer‐provided wide‐field calibration (WF method)

**FIGURE 2:**
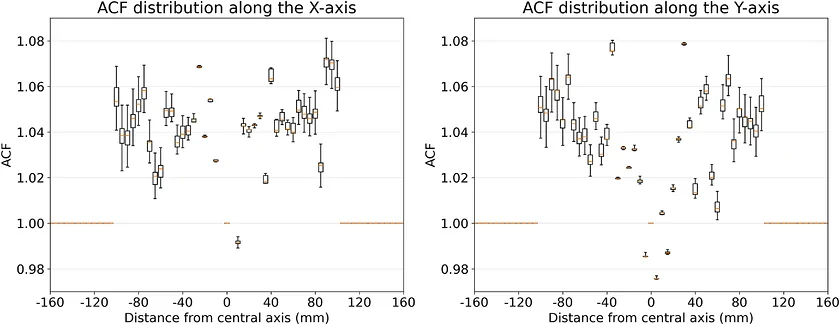
Box plots of the ACF distributions derived by the WF calibration method for individual detectors across five beam energies (4, 6, 10 MV, 6 MV FFF, and 10 MV FFF), along the (left) X‐axis and (right) Y‐axis profiles. Each box shows the five per‐energy ACF values of a single detector (center line, median; box, interquartile range; whiskers, full range; no points omitted), with a common ordinate scale for both panels. The upper and lower extremes of each box were predominantly associated with the 10 MV FFF and 4 MV beams, respectively, with the largest inter‐energy spread (≤2.9%) toward the field edge.

The standard calibration procedure recommended by the manufacturer, known as the WF method, was performed to determine the ACFs for correcting the sensitivities of individual ionization chambers. The WF method requires six independent measurements as described in previous studies^8^: three at the central alignment with the array rotated at 0°, 90°, and 180°, and three additional measurements with the array physically shifted in the lateral, longitudinal, and/or diagonal directions. This method calculates relative sensitivity differences by utilizing spatial substitution, in which different detectors are positioned to measure the beam intensity at the same spatial coordinates, ensuring that the calibration remains valid even for non‐uniform or FFF beam profiles. Although a single calibration file is often applied across multiple energies for relative distribution analysis, in this study the WF calibration procedure was repeated for every energy under identical setup conditions (SSD, buildup depth, and field size) to ensure the highest possible accuracy for comparison. Figure 2 illustrates the distribution of ACFs for each detector, derived via the WF calibration method across five photon beam energies.

For each energy, the WF calibration was performed on a single, randomly selected linac (TB2). Three independent ICP measurement sessions—one per linac (TB1–TB3), each paired with a corresponding water‐scanner acquisition—were used to evaluate the WF method. The resulting ACFs were applied to the ICP measurement data, and the performance was evaluated by calculating the percentage relative difference between the calibrated ICP profiles and the reference water‐scanner profiles within the in‐field region (as defined in Section 2.2.3). From these per‐detector relative differences, the Mean Absolute Error (MAE), standard deviation (SD), and maximum/minimum values were derived. MAE and SD were computed separately for each combination of beam energy and profile axis within each session, and reported values represent the mean across all sessions and combinations. Additionally, deviations in symmetry and flatness were investigated. Agreement in beam symmetry between the ICP and the water scanner was additionally assessed using Bland–Altman analysis, with the difference defined as ICP − water‐scanner symmetry; the mean bias, the 95% limits of agreement, and their 95% confidence intervals were computed for each calibration method. Total calibration times were also recorded for each method to compare workflow practicability. Statistical comparison between the WF method and the machine‐integrated WP method was performed using paired t‐tests, after verifying normality of paired differences via the Shapiro‐Wilk test. Each paired observation corresponded to a unique combination of linac, beam energy, and profile axis (*n* = 30 per metric).

#### 2.2.2 Water‐profile (WP) calibration method

**FIGURE 3:**
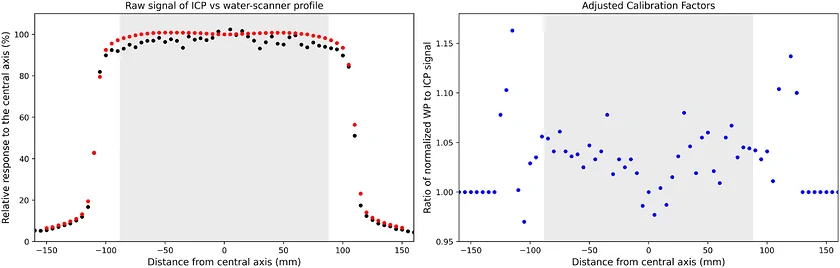
Representative profiles used for the derivation of ACFs using the proposed WP method, shown for the 10 MV beam on TB1 along the inline (Y) axis. (Left) The raw response signal measured by the ICP (black) and the reference dose profile measured by a water scanner (red) in a water phantom, and (Right) the resulting ACFs calculated by the ratio of the normalized water‐scanner profile to the normalized ICP signal. All profiles are shown as a function of distance from the central axis. The larger ACF excursions in the penumbra reflect the steep dose gradient and low signal there, consistent with the 10% CAX cutoff applied during ACF derivation (Section 2.2.2). The shaded band indicates the in‐field region (± 80% of the half field width).

The proposed WP method generates ACFs by directly matching ICP detector responses to the reference profiles measured by a 3D water scanner. The water‐scanner profiles, normalized to the central axis, were binned at 1 mm intervals and interpolated. Subsequently, 63 or 65 points were sampled at 0.5 cm intervals to correspond with the physical locations of the ionization chambers along the X (crossline) and Y (inline) axes of the ICP, respectively. The ACF for detector i($ACF_{i}$) is defined as the ratio of the normalized water‐scanner profile to the normalized ICP signal at that detector position:

$$\mathrm{AC}F_{i}=\frac{D_{\mathrm{WP},i}/D_{\mathrm{WP},\mathrm{CAX}}}{S_{\mathrm{ICP},i}/S_{\mathrm{ICP},\mathrm{CAX}}}$$

where $D_{WP,i}$ and $S_{ICP,i}$ denote the water‐scanner dose and ICP signal at detector position i, respectively, and $D_{WP,\mathrm{CAX}}$ and $S_{ICP,\mathrm{CAX}}$ are the corresponding values at the central axis (Figure 3). It should be noted that the sensitive volume of the CC13 reference chamber (0.13 cc) exceeds that of an individual ICP chamber (0.05 cc) by approximately a factor of 2.6; the implications of this mismatch for the WP‐derived ACF are discussed in Section 4. It is important to note that the ACF defined here represents the combined effect of two contributions: (i) the intrinsic sensitivity variation among individual ionization chambers (arising from electrical, geometric, and manufacturing differences), and (ii) any residual mismatch between the ICP raw response and the reference water profile at each detector position, which may include beam‐shape‐dependent detector response, volume averaging effects, and spatial sampling differences. Under the assumption that the intrinsic sensitivity variation is the dominant contributor, the WP‐derived ACF can be used to correct ICP measurements across different sessions of the same beam energy. This assumption is empirically tested in Section 2.2.3 through cross‐energy applicability testing.

ACFs were derived only for detectors whose signal exceeded 10% of the CAX value; outside this region the low signal‐to‐noise ratio and the steep dose gradient make the ratio‐based ACF unreliable. Consistent with this, the ACFs within the penumbra region exhibited a noticeably larger excursion than those in the in‐field region (see Figure 3), reflecting the same gradient‐driven sensitivity that causes the inter‐energy spread of ACFs to widen away from the central axis in Figure 2.

By definition, the WP‐derived ACF is a composite correction factor: it encapsulates not only the intrinsic sensitivity variation among detectors but also position‐dependent effects such as volume‐averaging differences between the reference (CC13) and ICP chambers, scatter and buildup differences between the water and slab phantom geometries, and residual setup mismatch between the two measurement systems.

The WP method was evaluated using two different data aggregation strategies to compare their effectiveness in reducing uncertainties associated with setup errors and machine‐specific characteristics, as well as their practicability for routine clinical implementation, including the time required for calibration. In the machine‐specific calibration, the ACF was derived using the water‐scanner data from a specific linac (e.g., TB1) and applied exclusively to the ICP measurements taken from that same unit. In the machine‐integrated calibration, the ACF was derived from the averaged water‐scanner profiles of three linac units (TB1–TB3), and this integrated calibration was then applied to the ICP measurements of each individual linac, enabling assessment of whether averaging across machines reduces random session‐to‐session setup variability.

#### 2.2.3 Evaluation of cross‐energy applicability

In this section, we investigated the feasibility of using a common calibration file across different beam energies. Because the position‐dependent contributions described in Section 2.B.2 can differ between beams of different energies, WP‐derived ACFs may not be fully invariant across energies, and a calibration file generated at one energy is not guaranteed to remain valid when applied to another.

To test this hypothesis, calibration files generated at each of the five nominal energies (4, 6, 10 MV flattened; 6, 10 MV FFF) were systematically cross‐applied to all other energies, yielding 20 cross‐energy combinations (5 calibration energies × 4 different evaluation energies). For instance, the ACF derived from 4 MV data was applied to evaluate measurements taken at 6 MV, 10 MV, 6 MV FFF, and 10 MV FFF. Self‐evaluation cases (matching calibration and evaluation energies) were excluded as they correspond to the same‐energy WP analysis. The cross‐evaluation was performed using an independent measurement session on TB1, with calibration files derived from a separate session on the same unit, to analyze whether energy‐specific calibration is a strict requirement for clinical accuracy. In addition to the overall analysis, sub‐group analyses were performed to compare same‐beam‐type transfers (FF↔FF and FFF↔FFF) with cross‐beam‐type transfers (FF↔FFF), examining whether substantial differences in beam profile shape between flattened and FFF beams affect the applicability of the WP‐derived ACF.

## 3 RESULTS

### 3.1 Performance of the WF method

When calibrated with the WF method, the overall MAE of the per‐detector relative differences was 0.31%, with a SD of 0.33%. The relative differences across all detectors ranged from −1.34% to +1.87%. Regarding the beam profile characterization metrics, the MAE for symmetry and flatness differences was 0.49% and 0.44%, respectively. These results, categorized by beam type (flattened vs. FFF), are summarized in Table 2. The WF method showed relatively higher point‐wise fluctuations compared to the proposed WP methods described in the following sections. The WF method required approximately 75 min (15 min × 5 energies, including array repositioning).

**TABLE 2:** Performance of the WF calibration method. Reported values are statistics of the per‐detector relative differences and the MAE of beam symmetry and flatness, pooled across three measurement sets (one per linac, TB1–TB3).

Metric (%)	All energies	Flattened	FFF
MAE of relative diff.	0.31	0.23	0.42
SD of relative diff.	0.33	0.26	0.44
Range of relative diff.	−1.34 to +1.87	−1.34 to +1.04	−0.80 to +1.87
MAE of symmetry diff.	0.49	0.51	0.47
MAE of flatness diff.	0.44	0.39	0.51

### 3.2 Performance of the WP method

For the machine‐specific calibration, in which the ACFs were derived from the TB1 water‐scanner profiles, the MAE and SD of the per‐detector relative differences were 0.15% and 0.15%, respectively, with a range from −0.70% to +0.77%. The MAE values for symmetry and flatness differences were 0.27% and 0.28%, respectively.

**FIGURE 4:**
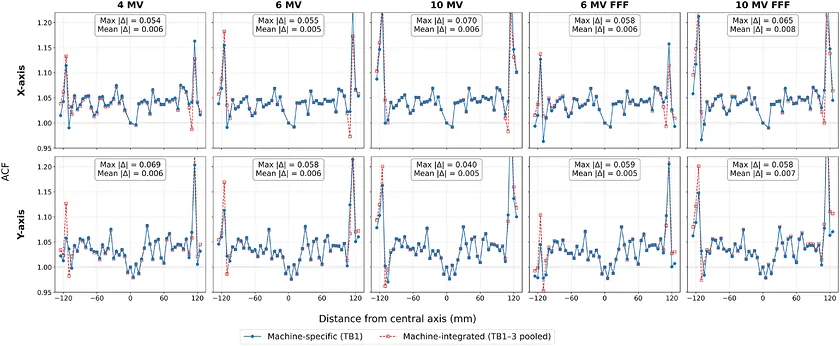
Per‐detector ACFs derived by the Water‐Profile (WP) method for five beam energies. Machine‐specific ACFs (blue circles; TB1) are overlaid with machine‐integrated ACFs (red squares; TB1–3 pooled). Top row: X‐axis; bottom row: Y‐axis. Insets show maximum and mean |ΔACF| between the two methods. Only detectors included in the ACF derivation (signal > 10% of the CAX value) are shown.

**FIGURE 5:**
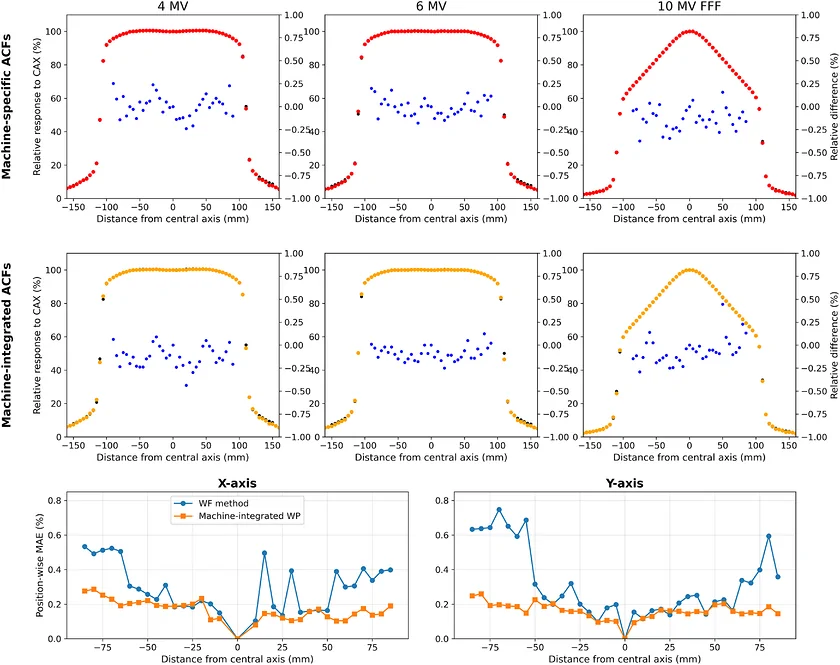
(Upper two rows) Beam profiles measured by the ICP and calibrated using machine‐specific (first row, red) and machine‐integrated (second row, orange) ACFs, overlaid with the corresponding water‐scanner reference profiles (black), for 4 MV (left), 6 MV (middle), and 10 MV FFF (right). Per‐detector relative differences within the in‐field region are shown on the right axis (blue). (Bottom row) Position‐wise MAE with respect to the water‐scanner reference for the WF (blue) and machine‐integrated WP (orange) methods along the X‐ and Y‐axes, computed from the same evaluation observations (three matched sessions × five energies; *n* = 15 per position, |x| ≤ 85 mm). The MAE at the central axis is zero by construction owing to the CAX normalization.

For the machine‐integrated calibration, in which the ACFs were derived from the averaged water‐scanner profiles of the three linacs (TB1–TB3), the MAE and SD of the per‐detector relative differences were 0.16% and 0.14%, respectively, with a range from −0.97% to +0.76%. The MAE values for symmetry and flatness differences were 0.21% and 0.27%, respectively. The WP method required approximately 10–15 minutes total. Figure 4 shows that the two variants produced essentially identical per‐detector ACFs across all five energies, and the per‐detector accuracy and beam profile characterization metrics were comparable between the two approaches (Table 3). The prominent spikes in the ACF curves of Figure 4 occur in the penumbra region, where steep dose gradients and low detector signals magnify small spatial mismatches between the ICP and the water‐scanner reference. The spatial distribution of the residuals across detector position is illustrated in Figure 5. To examine where the improvement occurs, the position‐wise MAE with respect to the water‐scanner reference was computed for the WF and machine‐integrated WP methods using the same evaluation observations (Figure 5, bottom row). The improvement was not uniform across the field: the two methods were comparable near the central axis, whereas toward the field periphery the WF position‐wise MAE increased to 0.36% (X‐axis) and 0.44% (Y‐axis) on average, with maxima of 0.53% and 0.75%, while the machine‐integrated WP error remained below 0.29% at all positions, indicating that the reduction achieved by the WP method is concentrated in the off‐axis region.

**TABLE 3:** Comparison of calibration performance across all evaluated methods.

Metric (%)	WF method	Machine‐specific	Machine‐integrated	Cross‐energy
MAE of relative diff.	0.31	0.15	0.16	0.25
SD of relative diff.	0.33	0.15	0.14	0.24
Range of relative diff.	−1.34 to +1.87	−0.70 to +0.77	−0.97 to +0.76	−1.02 to +1.02
MAE of symmetry diff.	0.49	0.27	0.21	0.43
MAE of flatness diff.	0.44	0.28	0.27	0.32

Paired *t*‐tests confirmed that the machine‐integrated WP method significantly reduced calibration uncertainties across all three metrics, with the normality assumption verified for each via the Shapiro‐Wilk test (*p* > 0.05 for all paired differences). The most pronounced improvement was in the per‐detector MAE, which decreased by 0.15% on average (*p* < 0.001, Cohen's *d* = 0.99, large effect). The absolute symmetry difference and the absolute flatness difference were also significantly reduced by 0.28% and 0.17%, respectively, with medium effect sizes (Cohen's *d* = 0.55 and 0.57; both *p* < 0.01). The analysis included 30 paired observations per metric, each corresponding to a unique combination of linac, beam energy, and profile axis.

### 3.3 Cross‐energy applicability of the WP method

The feasibility of using an ACF generated from one energy for beams of different energies was investigated across 20 cross‐energy combinations (5 calibration energies × 4 different evaluation energies) on TB1. When cross‐applying ACFs (e.g., applying a 6 MV ACF to a 10 MV profile), the overall MAE of the per‐detector relative differences was 0.25% and SD was 0.24%, with a range from −1.02% to +1.02%. The MAE for symmetry and flatness differences was 0.43% and 0.32%, respectively.

**FIGURE 6:**
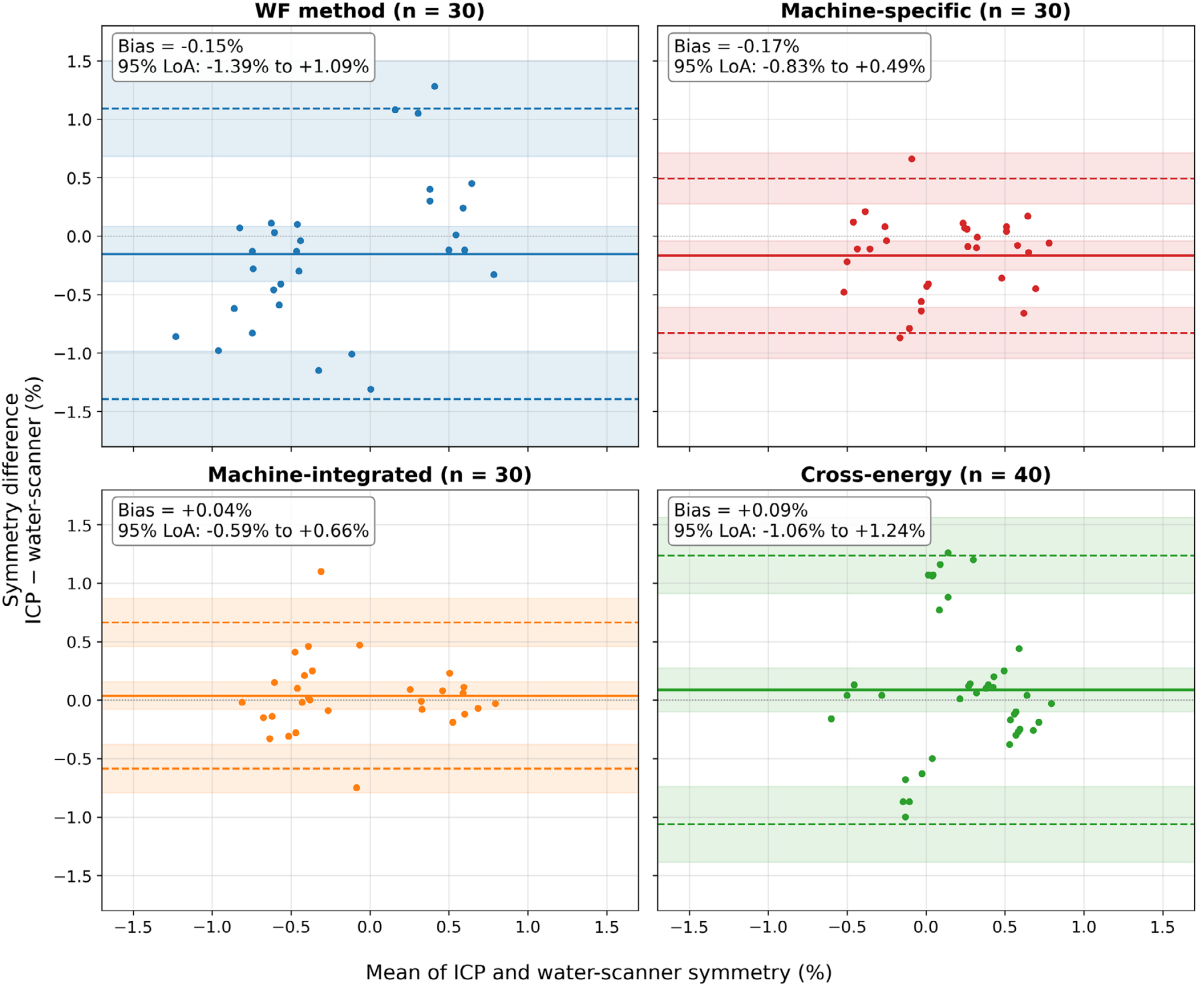
Bland–Altman plots of the beam symmetry agreement between the ICP and the water scanner for the four calibration methods: WF (top‐left, blue), machine‐specific WP (top‐right, red), machine‐integrated WP (bottom‐left, orange), and cross‐energy WP (bottom‐right, green). The difference is defined as ICP − water‐scanner symmetry and is plotted against the mean of the paired measurements. Each point represents one paired observation (a unique combination of linac, beam energy, and profile axis; *n* = 30 per panel), except for the cross‐energy panel, where each point corresponds to one of the 20 calibration–evaluation energy combinations per profile axis on TB1 (*n* = 40). Solid lines denote the mean bias, dashed lines the 95% limits of agreement (mean ± 1.96 SD), and shaded bands the 95% confidence intervals of these estimates.

Sub‐group analysis comparing same‐beam‐type transfers (FF↔FF and FFF↔FFF, *n* = 8 combinations) with cross‐beam‐type transfers (FF↔FFF, *n* = 12 combinations) yielded mean MAE values of 0.26% and 0.23%, respectively; the difference between same‐beam‐type and cross‐beam‐type transfers was small in this dataset. These cross‐energy values were higher than those of the energy‐specific WP calibration (MAE 0.15–0.16%) and lower than the corresponding WF value (MAE 0.31%) in this dataset. All quantitative metrics for both WP strategies are summarized in Table 3. Bland–Altman analysis of the symmetry agreement between the ICP and the water‐scanner measurements for the four calibration methods is shown in Figure 6. The mean bias (95% limits of agreement) was −0.15% (−1.39% to +1.09%) for the WF method, −0.17% (−0.83% to +0.49%) for the machine‐specific WP method, +0.04% (−0.59% to +0.66%) for the machine‐integrated WP method, and +0.09% (−1.06% to +1.24%) for the cross‐energy application.

## 4 DISCUSSION

The results of this study indicate that the proposed WP method provides both higher accuracy and a shorter calibration time than the manufacturer‐provided WF method. Paired statistical testing (*n* = 30) confirmed that the machine‐integrated WP method significantly reduced per‐detector MAE, symmetry differences, and flatness differences compared with the WF method (all *p* < 0.01), with large to medium effect sizes (Cohen's *d* = 0.55–0.99). The use of water‐scanner reference profiles acquired for WP calibration addresses the limitations of the generalized, geometry‐based WF calibration. It should be noted that the WP method aligns the ICP response to a measurement‐system‐specific reference (CC13/Blue Phantom2); it does not establish the true beam fluence, and closer agreement with this reference does not by itself demonstrate clinically superior QA performance.

The present work extends the concept introduced by Shen et al.^6^ along three directions that are relevant to conventional multi‐linac clinical environments. First, whereas Shen et al. investigated a single MR‐linac system with a single beam energy (7 MV FFF), the present work evaluates five photon energies (4, 6, 10 MV flattened; 6, 10 MV FFF) across three conventional C‐arm linacs. Second, we introduce and compare two reference‐data grouping strategies—machine‐specific and machine‐integrated—and show that a pooled reference derived from multiple units achieves accuracy comparable to unit‐specific calibration, which is of practical relevance for multi‐linac facilities. Third, the cross‐energy applicability of the WP‐derived ACFs is characterized through 20 cross‐energy combinations, including transfers between flattened and FFF beam types. These extensions can move the WP concept from a feasibility demonstration on a single MR‐linac toward a calibration strategy applicable to conventional radiation therapy clinics.

A relevant observation concerns the magnitude of the WF residual errors. The WF method yielded an average MAE of 0.49% for symmetry and 0.44% for flatness, with the maximum symmetry difference reaching 1.31% (observed in the 6 MV FFF beam, Y‐axis). These WF‐induced calibration uncertainties are of similar magnitude to the symmetry and flatness changes that routine QA workflows are designed to detect, indicating that the choice of calibration method can meaningfully influence the reliability of subsequent QA measurements. In contrast, the machine‐integrated WP method reduced these errors to 0.21% for symmetry and 0.27% for flatness, providing a larger margin between calibration‐induced uncertainty and the deviations that QA measurements are intended to detect. The smaller residuals observed with WP indicate closer agreement with the water‐scanner reference under the tested conditions. However, this study did not evaluate whether these differences would alter clinical QA pass/fail decisions, nor does it establish a clinical action threshold.

From a practical workflow perspective, the WP method offers a substantial advantage in calibration time. The WF process requires six independent irradiations with physical array repositioning (rotations and shifts) per energy, totaling approximately 75 min for five beam energies. The WP method, in contrast, requires only a single ICP irradiation per energy—approximately 10–15 min total when including initial setup. In this study, the water‐scanner reference profiles were acquired specifically for WP calibration, which requires a separate water‐scanner session. In practice, this acquisition burden can be mitigated by scheduling the ICP measurement immediately after a routine water‐scanner session, so that the reference profiles are obtained without an additional scanner setup. The subsequent ACF computation using in‐house scripts takes only a few seconds. This reduction in calibration time allows medical physicists to maintain calibration accuracy without long setup burdens throughout the clinical lifecycle of the linac.

Regarding the data grouping strategies, the machine‐specific and machine‐integrated approaches produced essentially equivalent per‐detector ACFs and calibration accuracy (Figure 4 and Table 3). Despite this equivalence in accuracy, the machine‐integrated approach can be considered preferable for two reasons. First, the pooled reference is obtained from three independent water‐scanner sessions, so the influence of random setup variations of any individual session is reduced. Second, the use of a pooled reference avoids the arbitrary selection of a single unit as the reference machine. A single pooled calibration file additionally simplifies the operational workflow, as a per‐machine calibration file does not need to be managed. For single‐linac institutions that wish to benefit from averaging out random setup variations, multiple independent measurement sets can be acquired and averaged to derive the calibration data.

In the investigation of cross‐energy applicability, applying an ACF derived from one energy to other energies yielded a per‐detector MAE of 0.25% and a symmetry MAE of 0.43%, higher than those of energy‐specific WP calibration (0.15–0.16% and 0.21–0.27%, respectively) and lower than the corresponding WF values (0.31% and 0.49%) in this dataset. Energy‐specific WP calibration remained more accurate, indicating the importance of matching the detector response to the actual beam profile of that specific energy. Sub‐group analysis showed comparable performance for same‐beam‐type (FF↔FF and FFF↔FFF) and cross‐beam‐type (FF↔FFF) transfers in this dataset. For clinical practice, energy‐specific ACF generation is therefore recommended whenever feasible, particularly when both flattened and FFF beams are in clinical use on the same linac.

The present study has several limitations. The evaluation was performed with a fixed 20 × 20 cm^2^ field size. The demonstrated validity of the WP‐derived ACFs is therefore limited to measurements performed at the same 20 × 20 cm^2^ field size as the calibration field; application to other field sizes, including the 30 × 30 cm^2^ fields used in annual QA, requires separate validation. The performance of the WP method may vary with field size for two reasons. First, for smaller fields (e.g., 5 × 5 or 10 × 10 cm^2^), the penumbra region occupies a larger fraction of the active detector array, which may amplify the volume‐averaging mismatch between the reference chamber and the ICP microchambers.^9^ Second, for larger fields (30 × 30 cm^2^ or greater), off‐axis beam softening and horn effects become more pronounced, which may alter the energy spectrum seen by peripheral detectors and modify the effective ACF. In contrast, the WF method is theoretically less sensitive to field size because it derives sensitivity differences through spatial substitution rather than through absolute profile matching. Systematic validation across clinically relevant field sizes (5 × 5 to 30 × 30 cm^2^) is therefore warranted. Future work will focus on validating the effectiveness of the WP method across various field sizes and on investigating its long‐term stability over multiple years to ensure that the ACFs remain valid as the detectors age. In addition, the reported numerical results are specific to the tested combination of TrueBeam linacs, the ICP, and the CC13/Blue Phantom2 water‐scanner system. Other institutions or device/reference‐chamber combinations should adapt the workflow to their own array geometry and water‐scanner reference data and perform local validation, rather than transferring the reported numerical performance directly. Furthermore, the observed residual differences comprise combined contributions from setup and positioning, water‐scanner measurement uncertainty, ICP repeatability and noise, and interpolation and sampling; the present design does not allow these components to be quantified separately, and the observed reduction therefore cannot be attributed to any single error component.

It should be emphasized that the WP‐derived ACF is, by construction, a composite correction factor that incorporates intrinsic detector sensitivity together with phantom geometry, volume‐averaging, and residual setup effects. The volume‐averaging contribution arises in part from the difference in spatial resolution between the reference CC13 chamber (0.13 cc) and the ICP microchambers (0.05 cc), which becomes appreciable in regions of steep dose gradient. This composite nature is the source of the method's strong per‐energy accuracy but also imposes the observed limitation on cross‐energy transferability. Approaches that attempt to isolate the intrinsic sensitivity component—for example, by applying Monte Carlo–based volume‐averaging corrections to the reference profile prior to ACF derivation—constitute a natural direction for future work aimed at improving cross‐energy robustness.

## 5 CONCLUSION

The present study developed and evaluated a water‐profile‐based calibration method (WP method) for 2D ionization chamber arrays used in linac QA. For the tested 20 × 20 cm^2^ field on three TrueBeam linacs with a CC13/Blue Phantom2 water‐scanner reference, the WP method provided closer agreement with the water‐scanner reference profiles than the manufacturer‐provided WF method: the MAE of the beam symmetry difference was reduced from 0.49% (WF) to 0.27% (machine‐specific WP) and 0.21% (machine‐integrated WP), corresponding to a 45–57% reduction; the MAE of the beam flatness difference was similarly reduced from 0.44% (WF) to 0.27–0.28% (WP); and the MAE of the per‐detector relative differences was reduced from 0.31% to 0.15–0.16%. In addition, the calibration time was substantially shortened. Rather than a universally superior replacement for the WF method, the WP method is presented as a calibration framework demonstrated for the tested configuration; the demonstrated validity is limited to the 20 × 20 cm^2^ calibration field, and application to other field sizes, including the 30 × 30 cm^2^ fields used in annual QA, requires separate validation. Institutions may adapt the framework to their own field sizes, device geometries, and water‐scanner reference data, followed by local validation.

## AUTHOR CONTRIBUTIONS

Author contributions are described using the CRediT (Contributor Roles Taxonomy). **Kihong Pak**: Conceptualization; methodology; software; validation; formal analysis; investigation; resources; data curation; writing—original draft; writing—review & editing; visualization; funding acquisition. **Sung‐woo Kim**: Investigation; data curation; funding acquisition. **Byungchul Cho**: Investigation; data curation. **Jungwon Kwak**: Investigation; data curation. **Seonghoon Jeong**: Investigation; data curation. **Kyeongyun Park**: Investigation; data curation. **Jisoo Kim**: Investigation; data curation. **Jonghan Park**: Investigation; data curation. **Si Yeol Song**: Investigation; data curation. **Seonyeong Noh**: Investigation; data curation. **Jongin Oh**: Investigation; data curation. **Seokjin Kang**: Investigation; data curation. **Chiyoung Jeong**: Conceptualization; methodology; software; validation; formal analysis; investigation; resources; data curation; visualization; supervision; project administration. All authors have read and approved the final manuscript.

## CONFLICT OF INTEREST STATEMENT

The authors declare no conflict of interest.

## ETHICS APPROVAL

This study did not involve human subjects or animal experiments; institutional review board approval was therefore not required.

## USE OF AI TOOLS

Generative AI tools (Anthropic Claude) were used in the preparation of this manuscript for the following limited purposes: (i) English‐language editing of author‐drafted text; (ii) reformatting and presentation of data that had already been analyzed by the authors; and (iii) independent cross‐verification of statistical test results that were primarily computed in Microsoft Excel. The AI tools were not used for primary data analysis, generation of figures, or interpretation of results. All scientific content, analyses, and conclusions are the work of the authors, who reviewed and verified every AI‐assisted output.

## Data Availability

The data that support the findings of this study are available from the corresponding author upon reasonable request.
